# Factors associated with concurrent cardiovascular disease in patients with rheumatoid arthritis and development of machine learning models for comorbidity identification

**DOI:** 10.3389/fcvm.2026.1860714

**Published:** 2026-08-19

**Authors:** Xinyan Shen, Tong Wang, Yi Guan

**Affiliations:** 1 College of Traditional Chinese Medicine, Beijing University of Chinese Medicine, Beijing, China; 2 Department of Traditional Chinese Medicine, Beijing Jishuitan Hospital, Capital Medical University, Beijing, China

**Keywords:** cardiovascular disease, comorbidity identification, cross-sectional study, machine learning, rheumatoid arthritis

## Abstract

**Objective:**

To analyze factors associated with concurrent cardiovascular disease (CVD) in patients with rheumatoid arthritis (RA) and to develop machine learning models for identifying current CVD comorbidity status using routinely available clinical data.

**Methods:**

This was a cross-sectional observational study. We included 4,767 patients with RA who attended Beijing Jishuitan Hospital from January 2021 to December 2024 and met the diagnostic criteria. According to the current presence of CVD, patients were divided into an RA-CVD group (*n* = 366) and an RA-non-CVD group (*n* = 4,401). Demographic characteristics, comorbidities, and laboratory indicators were collected. Univariate analysis was used to compare baseline characteristics between groups. Multivariable logistic regression models were then built after considering data completeness, clinical relevance, and collinearity. A high-completeness primary model was used as the main analysis, and a clinically enhanced model was used as a supplementary analysis. Multiple imputation was further performed as a missing-data sensitivity analysis to assess robustness. A simplified conventional-risk baseline model was additionally constructed using available traditional cardiovascular risk factors as a benchmark. Based on clinical data, we then developed core-variable models, wide-feature models, threshold-adjusted wide-feature models, and medication-information-available subgroup models. The dataset was divided into training and test sets at a ratio of 8:2, and 5-fold stratified cross-validation was performed in the training set. Model performance was evaluated by the area under the receiver operating characteristic curve (AUC), area under the precision-recall curve (PR-AUC), accuracy, sensitivity, specificity, and F1 score. The modeling task was defined as identification of concurrent CVD status rather than prediction of incident cardiovascular events.

**Results:**

The current prevalence of CVD in patients with RA was 7.68%. Compared with the RA-non-CVD group, the RA-CVD group had a higher proportion of men, older age, longer disease duration, and higher rates of hypertension and diabetes. Several indicators reflecting inflammatory burden, renal function, metabolic status, and hematologic characteristics also differed significantly between groups. Multivariable logistic regression showed that age, male sex, and disease duration were stable factors associated with CVD in RA in the high-completeness primary model. Multiple imputation sensitivity analysis further supported the robustness of these findings. In the clinically enhanced model, age and male sex remained stably associated with CVD, whereas hypertension, diabetes, erythrocyte sedimentation rate, and estimated glomerular filtration rate did not show stable independent statistical associations. The simplified conventional-risk baseline model showed comparable discrimination to the core-variable logistic regression model, but its sensitivity was very low at the default threshold of 0.50. The non-resampled baseline model achieved an AUC of 0.78 and a PR-AUC of 0.22, with a sensitivity of 0.04 at the default threshold. Core-variable models had moderate ability to distinguish patients with and without concurrent CVD. Adding a broader set of structured variables improved model discrimination, but sensitivity remained very low at the default threshold of 0.50. Training-derived Youden-threshold adjustment improved minority-class identification. For example, the sensitivity of the wide-feature XGBoost model increased from 0.06 at the default threshold to 0.81 after training-derived threshold adjustment.

**Conclusion:**

The current presence of CVD in RA reflects a complex comorbidity pattern characterized by multiple associated clinical factors. Age, male sex, and longer disease duration were relatively stable associated factors. Within the present internally validated setting, machine learning models based on routinely available clinical data may provide preliminary support for identifying concurrent CVD status in patients with RA. Expanding the variable set and applying training-derived threshold adjustment may improve recognition of minority-class patients in this imbalanced dataset. These findings provide a preliminary data-driven framework for comorbidity-oriented cardiovascular assessment in RA, but model stability, threshold transportability, and generalizability require further validation in multicenter prospective cohorts.

## 1 Introduction

Rheumatoid arthritis (RA) is a systemic autoimmune disease. Its main feature is chronic erosive arthritis. The main pathological change is chronic inflammation of the joint synovium. The resulting pannus damages cartilage and bone and finally leads to joint deformity and functional impairment (1). In addition to joint damage, RA can also affect multiple organs and systems, including the cardiovascular system. It is one of the major causes of disability and premature death in these patients. Epidemiological studies have shown that the risk of cardiovascular disease (CVD) in patients with RA is 1.5 to 2 times that of the general population, and the mortality rate can reach 40% to 50% (2). Therefore, clinical management of RA should not focus only on joint inflammation and functional impairment, but should also include timely recognition of cardiovascular comorbidity and comprehensive cardiovascular assessment (3).

The mechanism of RA combined with CVD is complex. It results from the combined effects of persistent inflammation, immune abnormalities, vascular endothelial injury, and metabolic disorders (4). The central pathological basis of RA is sustained activation of chronic immune inflammation. Proinflammatory pathways such as TNF-α, IL-6, and IL-1 remain highly activated for a long time. These same pathways also participate in the development and progression of atherosclerosis. This creates a shared inflammatory pathway between RA and CVD (5). In addition, persistent inflammation can cause endothelial dysfunction, increased oxidative stress, and upregulation of adhesion molecules. These changes promote the recruitment of monocytes and macrophages to the vascular wall. As a result, systemic inflammation gradually turns into local vascular inflammation (4). On this basis, RA-related inflammation can further disrupt lipid metabolism and lipoprotein function. This leads to the ‘lipid paradox.’ In other words, some patients do not show markedly elevated lipid levels, but their cardiovascular risk is still increased (4). Studies have shown that the anti-inflammatory and antioxidant functions of high-density lipoprotein are reduced in patients with RA. This weakens its protective role in reverse cholesterol transport (6). At the same time, increased serum cholesterol loading capacity can promote macrophage foam cell formation and is associated with the progression of coronary atherosclerosis in RA patients (7). In addition to large-vessel atherosclerosis, RA patients may also have coronary microvascular dysfunction and subclinical myocardial injury. This provides another important pathological pathway for cardiovascular involvement (8). Moreover, older age, male sex, hypertension, diabetes, dyslipidemia, and persistently high inflammatory burden can further amplify these pathological processes. This eventually increases the risk of coronary heart disease, stroke, and other CVD events (9).

Previous studies have compared the performance of QRISK-3, the Framingham Risk Score (FRS), and the Reynolds Risk Score (RRS) in RA, psoriasis (including psoriatic arthritis and psoriasis), and ankylosing spondylitis (AS). These findings suggest that cardiovascular assessment in RA cannot simply be transferred from general-population risk frameworks (10). In cross-sectional real-world data, an alternative and clinically relevant task is to characterize factors associated with existing CVD comorbidity and to examine whether routinely collected clinical variables can help identify concurrent CVD status. Machine learning offers a new way to integrate multidimensional variables, such as demographic features, metabolic markers, inflammatory markers, and treatment information. Machine learning has also been increasingly applied in broader medical risk assessment and outcome stratification. For example, Asteris et al. applied heuristic and data-ensemble algorithms to COVID-19 pandemic modeling, severity prognosis, and patient-outcome assessment, illustrating the potential of computational models to integrate complex biomedical information and support clinical decision-oriented risk evaluation (11–13). These studies suggest that machine learning methods may provide useful analytical frameworks for heterogeneous clinical conditions, although their clinical value depends on appropriate validation, calibration, and interpretation in specific disease contexts. Studies on RA-related models and complication models are increasing. Recent original studies have begun to examine RA-related cardiovascular involvement using multivariable or machine-learning approaches. Feng et al. used univariate analysis, LASSO, random forest, and logistic regression to identify CVD-related indicators in patients with RA and further constructed individualized nomograms, supporting the potential value of multidimensional clinical and laboratory variables for RA-CVD assessment (14). In a Chinese RA cohort, Zou et al. compared several conventional cardiovascular risk models and showed that traditional risk frameworks require careful evaluation and calibration when applied to RA populations (15).

However, evidence specifically addressing concurrent CVD in patients with RA remains limited, particularly regarding the associated clinical profile and the value of available structured clinical data for comorbidity identification (16). Against this background, the present study used real-world data from 4,767 patients with RA to examine factors associated with concurrent CVD and to develop machine learning models for identifying current comorbidity status. The manuscript presents conventional statistical and sensitivity analyses of the associated factors, followed by a simplified conventional-risk reference analysis and the evaluation of machine learning models using different feature sets and decision thresholds, before discussing the clinical and methodological implications of the findings. By integrating associated-factor analysis with threshold-dependent model evaluation, this study extends the available evidence on the characteristics of patients with RA and concurrent CVD, while illustrating the potential value of available clinical data and the performance trade-offs associated with decision-threshold selection.

## 2 Materials and methods

### 2.1 Study population

This was a cross-sectional observational study. The study included patients who visited Beijing Jishuitan Hospital between January 2021 and December 2024 and met the diagnostic criteria for rheumatoid arthritis (RA). The inclusion criteria were as follows: (1) fulfillment of either the 1987 American College of Rheumatology classification criteria for RA or the 2010 ACR/EULAR classification criteria for RA; and (2) age ≥18 years. The exclusion criteria were as follows: (1) failure to meet the above inclusion criteria; (2) coexistence of other clearly diagnosed systemic rheumatic autoimmune diseases, active malignant tumors, or severe hepatic or renal dysfunction that might significantly affect the main study variables; and (3) missing key outcome information or inability to complete the main clinical assessment. A total of 4,767 patients were finally included. Among them, 366 had RA with CVD and 4,401 had RA without CVD.

### 2.2 Outcome definition

The outcome variable was the presence of concurrent CVD at the time of clinical assessment. CVD status was determined from medical records, previously confirmed diagnoses, and relevant examination results. This outcome reflected prevalent cardiovascular or cerebrovascular comorbidity rather than incident cardiovascular events during follow-up. It mainly included coronary heart disease, myocardial infarction, heart failure, stroke, hypertensive heart disease, peripheral arterial disease, and other clearly diagnosed cardiovascular or cerebrovascular diseases. According to the presence or absence of these conditions, the patients were divided into the RA with CVD group (RA-CVD group) and the RA without CVD group (RA-non-CVD group).

Because this study used a composite CVD outcome, the distribution of the recorded CVD subtypes was reported descriptively in Supplementary Table S1. These conditions were grouped to characterize the overall burden of clinically documented cardiovascular and cerebrovascular comorbidity and to retain sufficient outcome events for the main analyses. Separate subtype-specific models were not performed because several subtypes had limited event counts and the diagnostic categories were not mutually exclusive. Instead, a restricted-outcome sensitivity analysis focusing on major atherosclerotic cardiovascular or cerebrovascular diseases was conducted to examine whether the main associated-factor pattern was influenced by the inclusion of heterogeneous CVD components.

### 2.3 Study variables

Demographic data, disease duration, physical measurements, comorbidity information, and laboratory test results were collected. The main variables included in the conventional statistical analysis were age, sex, disease duration, body mass index (BMI), hypertension, diabetes, dyslipidemia, mean systolic blood pressure, mean diastolic blood pressure, erythrocyte sedimentation rate, C-reactive protein, rheumatoid factor, blood glucose, total cholesterol, triglycerides, low-density lipoprotein, high-density lipoprotein, homocysteine, uric acid, estimated glomerular filtration rate, white blood cell count, hemoglobin, platelet count, and neutrophil-to-lymphocyte ratio.

### 2.4 Conventional statistical analysis

Continuous variables with an approximately normal distribution were expressed as mean ± standard deviation (x¯ ± s), and comparisons between groups were performed using the independent-samples t test. Continuous variables with a non-normal distribution were expressed as median (quartiles) [M (Q1, Q3)], and comparisons between groups were performed using the Wilcoxon rank-sum test. Categorical variables were expressed as number and percentage, and comparisons between groups were performed using the chi-square test or Fisher's exact test.

Based on the univariate analysis, multivariable logistic regression models were constructed after considering variable completeness, clinical relevance, and collinearity diagnostics. To balance sample utilization and clinical interpretability, two models were built: a high-completeness primary model and a clinically enhanced model. The high-completeness primary model was used as the main inferential model, and the clinically enhanced model was used as a supplementary analysis model. The results were reported as odds ratios (ORs) with 95% confidence intervals (CIs). For continuous variables with small per-unit effects, ORs were expressed per clinically interpretable increments, as specified in the corresponding tables. Specifically, estimated glomerular filtration rate was reported per 10-mL/min/1.73 m^2^ increase, erythrocyte sedimentation rate and C-reactive protein per 10-unit increase, uric acid and platelet count per 50-unit increase. This rescaling changed only the unit used to interpret the effect estimates and did not affect model fit, *P* values, or statistical conclusions. ORs and 95% CIs were presented to two decimal places, whereas *P* values were presented to three decimal places, with values below 0.001 reported as *P* < 0.001.

To evaluate the effect of missing data on the multivariable analysis, a sensitivity analysis for missing values was further performed. Complete-case analysis was used as the main modeling strategy. Because complete-case analysis substantially reduced the analytic sample size, missingness rates of candidate variables were explicitly summarized and used to guide model specification. Multiple imputation by chained equations (MICE) was used to impute missing values in variables included in the multivariable regression analysis. A total of 20 imputed datasets were generated, with 20 iterations. The imputation model included the outcome variable and all covariates entered into the regression analysis, but the outcome variable itself was not imputed. Specifically, for the high-completeness primary model, the imputation procedure included CVD status, age, sex, disease duration, and erythrocyte sedimentation rate. For the clinically enhanced model, the imputation procedure included CVD status, age, sex, disease duration, hypertension, diabetes, erythrocyte sedimentation rate, and estimated glomerular filtration rate. Regression models were fitted separately in each imputed dataset, and the parameter estimates were combined using Rubin's rules. The combined results were then compared with the complete-case results to assess the consistency of effect directions and statistical significance. Complete-case results were interpreted together with multiple-imputation sensitivity analyses rather than as standalone definitive estimates. A two-sided *P* value <0.05 was considered statistically significant. The missingness profile of the main candidate clinical variables is shown in Supplementary Tables S2a, S2b.

To assess whether the main associated-factor pattern was influenced by clinically heterogeneous components of the composite CVD outcome, a restricted-outcome sensitivity analysis was further performed. The restricted outcome included coronary atherosclerotic disease-related diagnoses, myocardial infarction, stroke/cerebrovascular events, and peripheral arterial disease. These conditions were selected to construct a more vascular-focused outcome because they share more closely related pathological features involving vascular injury, endothelial dysfunction, chronic inflammation, and ischemic or thrombotic processes. Heart failure/cardiac insufficiency was excluded because it is a heterogeneous clinical syndrome that may result from multiple cardiac and systemic mechanisms. This analysis was intended to reduce, but not eliminate, heterogeneity in the composite outcome. The same high-completeness primary logistic regression model was refitted to examine whether the main associated-factor pattern remained consistent.

### 2.5 Machine learning analysis

The machine learning task was defined as identification of concurrent CVD status in patients with RA. The models were developed to recognize current cardiovascular comorbidity, not to predict incident cardiovascular events or infer causal risk. To provide a benchmark based on available conventional cardiovascular risk information, a simplified conventional-risk baseline model was additionally constructed. This model was not a direct comparison with validated cardiovascular risk scores. Because several variables required for formal scores such as QRISK-3 or the Framingham Risk Score were unavailable or highly incomplete, these scores could not be reconstructed in this dataset. Therefore, the baseline model used available traditional cardiovascular risk factors, including age, sex, hypertension, diabetes, dyslipidemia, smoking history, BMI, mean systolic blood pressure, and low-density lipoprotein cholesterol. The primary conventional-risk baseline model was fitted without synthetic minority over-sampling technique (SMOTE) to preserve its role as a conventional clinical reference. A SMOTE-based version was additionally evaluated as a supplementary comparison under the same class-imbalance handling strategy used in the core-variable machine learning models. The same training-test split, training-set preprocessing strategy, and evaluation metrics were used to ensure comparability across models.

In addition to this benchmark, four groups of machine learning analyses were designed in this study. In the first group, the core-variable models, and in the fourth group, the subgroup analysis with available medication information, identification models were built using logistic regression, LASSO, random forest, and XGBoost. In the second group, the wide-feature models, and in the third group, the wide-feature threshold-adjusted models, LASSO, random forest, and XGBoost were compared to evaluate identification performance under different feature sets and threshold settings.

The first group comprised the core-variable model. It included nine core variables selected from the univariate analysis and clinical relevance. These variables were age, sex, disease duration, hypertension, diabetes, erythrocyte sedimentation rate, estimated glomerular filtration rate, white blood cell count, and hemoglobin.

The second group was the wide-feature model. First, patient identifiers, text variables, and auxiliary fields that might cause information leakage were removed. Then, the remaining structured variables were screened automatically. Numeric variables were required to have a missing rate of no more than 95% and could not be completely missing or have only one unique value. Categorical variables were required to have a missing rate of no more than 95% and 2 to 10 categories. These screening rules were applied within the training data, and the final wide-feature set was determined accordingly. A total of 86 wide features were finally retained for model development. Details are shown in Supplementary Table S3.

The third group was the wide-feature threshold-adjusted model. Based on the second group, the Youden threshold was further determined using out-of-fold predicted probabilities from the training set. This was done to explore whether a training-derived threshold could better address class imbalance and improve identification of the minority CVD class.

The fourth group was the subgroup analysis with available medication information. Only patients with complete medication information were included. On the basis of the base feature model, one additional coarse-grained variable, ‘use of antirheumatic drugs,’ was added to explore the potential effect of medication-related information on model performance.

### 2.6 Data preprocessing and hyperparameter optimization

In all machine learning analyses, the random seed was set to 2026. After stratification by outcome, the data were split into a training set and a test set at a ratio of 8:2. Five-fold stratified cross-validation was performed in the training set. To avoid data leakage, missing value imputation, class resampling, standardization, feature encoding, and hyperparameter optimization were all performed only within the training set. The test set was used only for final independent evaluation.

In the core-variable models, missing values in continuous variables were imputed with the median, and missing values in categorical variables were imputed with the mode. One-hot encoding, removal of zero-variance variables, and standardization were then performed in sequence. In the SMOTE-based conventional-risk baseline model and the core-variable models, SMOTE was applied within the training set to reduce the dominance of the majority class and improve minority-class learning. The test set was not resampled and was used only for final independent evaluation.

In the wide-feature models, missing values in continuous variables were imputed with the median. Missing values in categorical variables were coded as ‘Unknown.’ New categories that appeared only in the test set were coded as ‘Novel,’ and low-frequency categories were merged into ‘Other.’ One-hot encoding, removal of zero-variance variables, and standardization were then performed. For hyperparameter optimization, LASSO used regular grid search for the penalty parameter. Random forest used a fixed number of 800 trees and searched for mtry and min_n. XGBoost also used a fixed number of 800 trees and applied random search to optimize tree depth, learning rate, minimum loss reduction, minimum number of samples in leaf nodes, sampling ratio, and mtry. For all models, the area under the receiver operating characteristic curve (AUC) from cross-validation was used as the main optimization metric.

To reduce the risk of overfitting and data leakage, all model-development procedures were restricted to the training set. Missing-value imputation, feature screening, categorical encoding, standardization, SMOTE, hyperparameter optimization, and training-derived threshold selection were performed without using test-set information. Hyperparameters were optimized by 5-fold stratified cross-validation within the training set, and the held-out test set was used only for final performance evaluation. For the threshold-adjusted wide-feature XGBoost model, which showed the most favorable numerical profile, model-complexity and regularization-related parameters, including tree depth, learning rate, minimum loss reduction, minimum number of samples in terminal nodes, sampling ratio, and mtry, were tuned within the training data. These procedures were used to reduce, rather than eliminate, the risk of overfitting in the internally developed models.

### 2.7 Model evaluation

Receiver operating characteristic (ROC) curves and AUC were used to evaluate model discrimination. PR-AUC was also reported to assess the identification of the minority class under class imbalance. Accuracy, sensitivity, specificity, positive predictive value, negative predictive value, and F1 score were calculated under both the default threshold of 0.50 and the training-derived Youden threshold. Calibration curves and the Brier score were used to assess the agreement between predicted probabilities and actual outcomes. For the main regression-based models, the Hosmer-Lemeshow test was further used to evaluate goodness of fit. Decision curve analysis (DCA) was used to explore the potential net benefit of different models across threshold ranges for concurrent CVD identification.

Model-performance estimates, including AUC, PR-AUC, Brier score, accuracy, sensitivity, specificity, positive predictive value, negative predictive value, F1 score, and decision thresholds, were reported to two decimal places. *P* values were reported to three decimal places, with values below 0.001 expressed as *P* < 0.001.

### 2.8 Ethics statement

The study protocol was approved by the Ethics Committee of Beijing Jishuitan Hospital, Capital Medical University (Approval No. Jilun [K2026] No. [044]-00). This was an observational study based on existing clinical data. All data were de-identified, and the requirement for informed consent was waived by the ethics committee.

## 3 Results

### 3.1 Comparison of baseline characteristics

A total of 4,767 patients with RA were included in this study. Among them, 366 patients had RA with CVD, accounting for 7.68%, and 4,401 patients had RA without CVD, accounting for 92.32%. The comparison of baseline characteristics between the two groups is shown in Table 1. Compared with the RA-non-CVD group, the RA-CVD group had a higher proportion of men, older age, a higher prevalence of hypertension and diabetes, longer disease duration, and higher mean systolic blood pressure. For laboratory indicators, the RA-CVD group had higher levels of erythrocyte sedimentation rate, blood glucose, uric acid, and white blood cell count than the RA-non-CVD group, while estimated glomerular filtration rate and hemoglobin levels were lower. All of these differences were statistically significant (all *P* < 0.05). There were no significant differences between the two groups in smoking history, dyslipidemia, BMI, mean diastolic blood pressure, C-reactive protein, rheumatoid factor, total cholesterol, triglycerides, low-density lipoprotein cholesterol, high-density lipoprotein cholesterol, homocysteine, platelet count, or neutrophil-to-lymphocyte ratio (all *P* > 0.05).

**Table 1:** Comparison of baseline characteristics between the RA-CVD group and the RA-non-CVD group.

Variable	RA-non-CVD group (*n* = 4,401)	RA-CVD group (*n* = 366)	*P* value
Sex [*n* (%)]			<0.001
Male	970 (22.0)	128 (35.0)	
Female	3,431 (78.0)	238 (65.0)	
Smoking history [*n* (%)]			0.861
No	419 (84.0)	86 (82.7)	
Yes	80 (16.0)	18 (17.3)	
Hypertension [*n* (%)]			0.001
No	1,230 (68.4)	192 (59.1)	
Yes	569 (31.6)	133 (40.9)	
Diabetes [*n* (%)]			0.030
No	1,577 (87.7)	270 (83.1)	
Yes	222 (12.3)	55 (16.9)	
Dyslipidemia [*n* (%)]			0.314
No	1,720 (95.6)	306 (94.2)	
Yes	79 (4.4)	19 (5.8)	
Age (years)	53.46 ± 14.44	66.84 ± 9.10	<0.001
Disease duration [years, M (Q1, Q3)]	6.00 (4.00, 11.00)	6.00 (4.00, 14.00)	0.012
BMI (kg/m^2^)	23.94 ± 3.89	24.32 ± 4.20	0.381
Mean systolic blood pressure (mmHg)	129.39 ± 15.53	137.94 ± 18.77	<0.001
Mean diastolic blood pressure (mmHg)	76.98 ± 9.57	77.85 ± 10.11	0.399
Erythrocyte sedimentation rate (mm/h) [M (Q1, Q3)]	24.00 (12.00, 45.00)	30.00 (15.00, 52.00)	<0.001
C-reactive protein (mg/L) [M (Q1, Q3)]	2.09 (0.50, 8.85)	2.62 (0.54, 11.75)	0.081
Rheumatoid factor (IU/mL) [M (Q1, Q3)]	76.80 (20.67, 249.25)	146.00 (34.92, 254.50)	0.381
Blood glucose (mmol/L)	5.23 ± 1.01	5.51 ± 1.49	<0.001
Total cholesterol (mmol/L)	4.94 ± 1.13	4.54 ± 1.28	0.120
Triglycerides (mmol/L) [M (Q1, Q3)]	1.14 (0.86, 1.71)	1.11 (0.92, 1.43)	0.937
Low-density lipoprotein cholesterol (mmol/L)	2.75 ± 0.87	2.61 ± 0.94	0.405
High-density lipoprotein cholesterol (mmol/L)	1.52 ± 0.47	1.53 ± 0.42	0.962
Homocysteine (μmol/L) [M (Q1, Q3)]	10.10 (7.88, 13.10)	13.60 (10.15, 15.85)	0.140
Uric acid (μmol/L) [M (Q1, Q3)]	272.50 (222.00, 339.00)	312.00 (238.50, 361.00)	0.001
Estimated glomerular filtration rate [mL/(min·1.73 m^2^)]	100.78 ± 17.79	88.73 ± 17.86	<0.001
White blood cell count ( × 10^9/L)	6.72 ± 2.22	7.76 ± 4.16	<0.001
Hemoglobin (g/L)	122.56 ± 17.05	117.42 ± 17.25	0.002
Platelet count ( × 10^9/L)	267.03 ± 82.31	271.28 ± 99.86	0.600
Neutrophil-to-lymphocyte ratio [M (Q1, Q3)]	2.38 (1.69, 3.46)	3.56 (2.08, 4.68)	0.057

Categorical variables are presented as n. Continuous variables are presented as mean ± standard deviation or median (quartiles), according to their distribution. Percentages for categorical variables were calculated based on the number of non-missing cases for each variable.

### 3.2 Univariate logistic regression analysis

The results of the univariate logistic regression analysis are shown in Table 2. The results showed that male sex, older age, longer disease duration, higher mean systolic blood pressure, hypertension, diabetes, higher erythrocyte sedimentation rate, higher C-reactive protein, higher blood glucose, higher uric acid, and higher white blood cell count were all associated with CVD in patients with RA (all *P* < 0.05). Among these factors, the OR for RA with CVD was 1.90 in men compared with women (95% CI: 1.51–2.38, *P* < 0.001). For each 1-year increase in age, the OR for RA with CVD was 1.08 (95% CI: 1.07–1.10, *P* < 0.001). For each 1-year increase in disease duration, the OR was 1.03 (95% CI: 1.02–1.04, *P* < 0.001). The ORs for RA with CVD were 1.50 in patients with hypertension (95% CI: 1.17–1.91, *P* = 0.001), 1.45 in patients with diabetes (95% CI: 1.04–1.98, *P* = 0.025), and 1.35 in patients with dyslipidemia (95% CI: 0.79–2.22, *P* = 0.252).

**Table 2:** Univariate logistic regression analysis of factors associated with CVD in patients with RA.

Variable	^*n*^	No. events	OR	95% CI	*P* value
Sex (Male vs. Female)	4,767	366	1.90	1.51–2.38	<0.001
Age (per 1-year increase)	4,767	366	1.08	1.07–1.10	<0.001
Disease duration (per 1-year increase)	4,164	327	1.03	1.02–1.04	<0.001
Mean systolic blood pressure (per 1-mmHg increase)	619	106	1.03	1.02–1.05	<0.001
Estimated glomerular filtration rate (per 10-mL/min/1.73 m^2^ increase)	2,266	170	0.73	0.67–0.78	<0.001
White blood cell count (per 1 × 10^9/L increase)	1,220	120	1.14	1.07–1.22	<0.001
Blood glucose (per 1-mmol/L increase)	2,408	184	1.21	1.08–1.35	<0.001
Erythrocyte sedimentation rate (per 10-mm/h increase)	2,886	237	1.07	1.03–1.13	0.001
Hypertension (Yes vs. No)	2,124	325	1.50	1.17–1.91	0.001
Hemoglobin (per 1-g/L increase)	1,220	120	0.98	0.97–0.99	0.002
Uric acid (per 50-μmol/L increase)	2,405	183	1.11	1.05–1.22	0.006
Diabetes (Yes vs. No)	2,124	325	1.45	1.04–1.98	0.025
C-reactive protein (per 10-mg/L increase)	1,495	137	1.07	1.00–1.14	0.036
Total cholesterol (per 1-mmol/L increase)	211	22	0.73	0.48–1.08	0.122
Rheumatoid factor (per 1-IU/mL increase)	398	34	1.00	1.00–1.00	0.133
Homocysteine (per 1-μmol/L increase)	103	7	1.08	0.95–1.20	0.199
Dyslipidemia (Yes vs. No)	2,124	325	1.35	0.79–2.22	0.252
BMI (per 1-kg/m^2^ increase)	611	102	1.02	0.97–1.08	0.381
Mean diastolic blood pressure (per 1-mmHg increase)	619	106	1.01	0.99–1.03	0.398
Low-density lipoprotein cholesterol (per 1-mmol/L increase)	249	34	0.84	0.54–1.27	0.404
Triglycerides (per 1-mmol/L increase)	213	22	0.82	0.43–1.24	0.446
Neutrophil-to-lymphocyte ratio (per 1-unit increase)	298	30	1.03	0.89–1.13	0.597
Platelet count (per 50 × 10⁹/L increase)	1,220	120	1.05	0.90–1.16	0.599
Smoking history (Yes vs. No)	603	104	1.10	0.61–1.89	0.749
High-density lipoprotein cholesterol (per 1-mmol/L increase)	210	22	1.02	0.37–2.57	0.962

*n* indicates the number of non-missing observations for that variable, and the number of events indicates the number of RA-CVD cases among the non-missing observations for that variable. For binary variables, ‘No’ or female was used as the reference category. For continuous variables, the OR represents the change in odds associated with the increment specified in the variable label. ORs and 95% CIs are presented to two decimal places, whereas *P* values are presented to three decimal places.

In contrast, a higher estimated glomerular filtration rate and higher hemoglobin were negatively associated with RA with CVD. For each 10-mL/min/1.73 m^2^ increase in estimated glomerular filtration rate, the OR for concurrent CVD was 0.73 (95% CI: 0.67–0.78, *P* < 0.001). For each 1-g/L increase in hemoglobin, the OR was 0.98 (95% CI: 0.97–0.99, *P* = 0.002). Smoking history, BMI, mean diastolic blood pressure, total cholesterol, rheumatoid factor, homocysteine, low-density lipoprotein cholesterol, high-density lipoprotein cholesterol, triglycerides, platelet count, and neutrophil-to-lymphocyte ratio were not significantly associated with RA with CVD (all *P* > 0.05).

### 3.3 Multivariable logistic regression analysis

The missingness profile of major candidate variables is summarized in Supplementary Tables S2a and S2b. Age and sex had complete data, and disease duration had relatively low missingness. In contrast, several lifestyle, physical measurement, comorbidity, and laboratory variables had substantial missingness. For example, smoking history, BMI, mean systolic blood pressure, and mean diastolic blood pressure had missing rates above 80%, whereas hypertension, diabetes, and estimated glomerular filtration rate had missing rates of 55.44%, 55.44%, and 52.46%, respectively. Therefore, the high-completeness primary model prioritized variables with relatively better availability and clinical relevance, whereas the clinically enhanced model was used as a supplementary model to examine additional clinically important but less complete variables.

Based on the univariate analysis, and after considering variable completeness, clinical relevance, and collinearity diagnostics, a high-completeness primary model and a clinically enhanced model were constructed. After considering model discrimination, data completeness, and the results of the missing-data sensitivity analysis, the high-completeness primary model was used as the main multivariable model in the main text, and the clinically enhanced model was used as a supplementary model. The results of the multivariable logistic regression analysis are shown in Table 3. The results of the missing-data sensitivity analysis are shown in Supplementary Tables S4 and S5.

**Table 3:** Multivariable logistic regression analysis of factors associated with CVD in patients with RA.

Model	Variable	Adjusted OR	95% CI	*P* value
High-completeness primary model (*n* = 2,629, No. events=219, No. non-events=2,410, AUC=0.78)	Age (per 1-year increase)	1.07	1.06–1.09	<0.001
	Sex (Male vs. Female)	2.20	1.63–2.98	<0.001
	Disease duration (per 1-year increase)	1.02	1.01–1.04	0.006
	Erythrocyte sedimentation rate (per 10-mm/h increase)	1.01	0.97–1.06	0.570
Clinically enhanced model (*n* = 914, No. events = 134, No. non-events = 780, AUC = 0.72)	Age (per 1-year increase)	1.06	1.03–1.08	<0.001
	Sex (Male vs. Female)	2.26	1.52–3.35	<0.001
	Disease duration (per 1-year increase)	1.01	0.99–1.03	0.459
	Hypertension (Yes vs. No)	0.90	0.60–1.35	0.612
	Diabetes (Yes vs. No)	0.79	0.43–1.44	0.447
	Erythrocyte sedimentation rate (per 10-mm/h increase)	1.04	0.98–1.11	0.231
	Estimated glomerular filtration rate (per 10-mL/min/1.73 m^2^ increase)	0.99	0.87–1.13	0.883

The high-completeness primary model included age, sex, disease duration, and erythrocyte sedimentation rate. The clinically enhanced model further included hypertension, diabetes, and estimated glomerular filtration rate. For continuous variables, the OR represents the change in odds associated with the increment specified in the variable label. Erythrocyte sedimentation rate is reported per 10-mm/h increase, and estimated glomerular filtration rate is reported per 10-mL/min/1.73 m^2^ increase. Adjusted ORs and 95% CIs are presented to two decimal places, whereas *P* values are presented to three decimal places.

The high-completeness primary model included 2,629 patients, including 219 patients with RA-CVD and 2,410 patients with RA-non-CVD. The model AUC was 0.78. The results showed that age, male sex, and disease duration remained independent factors associated with RA combined with CVD. For each 1-year increase in age, the OR for RA combined with CVD was 1.07 (95% CI: 1.06–1.09, *P* < 0.001). The OR for RA combined with CVD in men was 2.20 (95% CI: 1.63–2.98, *P* < 0.001). For each 1-year increase in disease duration, the OR was 1.02 (95% CI: 1.01–1.04, *P* = 0.006). After adjustment for other variables, erythrocyte sedimentation rate was no longer significantly associated with concurrent CVD (per 10-mm/h increase: OR = 1.01, 95% CI: 0.97–1.06, *P* = 0.570). In the sensitivity analysis using multiple imputation, age (OR = 1.08, 95% CI: 1.07–1.09, *P* < 0.001), male sex (OR = 1.72, 95% CI: 1.35–2.18, *P* < 0.001), and disease duration (OR = 1.01, 95% CI: 1.00–1.03, *P* = 0.026) remained statistically significant, and the effect directions were consistent with those in the complete-case analysis. Erythrocyte sedimentation rate also remained non-significant in the multiple-imputation analysis (per 10-mm/h increase: OR = 1.01, 95% CI: 0.96–1.06, *P* = 0.642). These findings suggest that the results of the high-completeness primary model were relatively robust.

The clinically enhanced model included 914 patients, including 134 patients with RA-CVD and 780 patients with RA-non-CVD. The model AUC was 0.72. The results showed that after further adding hypertension, diabetes, and estimated glomerular filtration rate, age and male sex still remained independently associated with RA combined with CVD. For each 1-year increase in age, the OR for RA combined with CVD was 1.06 (95% CI: 1.03–1.08, *P* < 0.001). The OR for RA combined with CVD in men was 2.26 (95% CI: 1.52–3.35, *P* < 0.001). In contrast, disease duration, hypertension, diabetes, erythrocyte sedimentation rate, and estimated glomerular filtration rate did not show independent statistical associations in this model (all *P* > 0.05). In the multiple-imputation sensitivity analysis, age (OR = 1.08, 95% CI: 1.06–1.09, *P* < 0.001) and male sex (OR = 1.72, 95% CI: 1.35–2.19, *P* < 0.001) remained significant. The independent association of disease duration in the clinically enhanced model showed some fluctuation between the complete-case analysis and the multiple-imputation analysis (OR = 1.02, 95% CI: 1.00–1.03, *P* = 0.022), which suggests that its effect estimate may have been influenced by data completeness and model specification. Hypertension, diabetes, erythrocyte sedimentation rate, and estimated glomerular filtration rate still did not show stable independent associations.

Collinearity diagnostics showed that the variance inflation factor ranges were 1.01–1.03 in the high-completeness primary model and 1.01–1.54 in the clinically enhanced model. All values were low, which suggests that there was no obvious multicollinearity in either model.

To examine whether the results were influenced by heterogeneity in the composite CVD outcome, a restricted-outcome sensitivity analysis was performed by limiting the outcome to major atherosclerotic cardiovascular or cerebrovascular disease. In the full cohort, 327 patients met the restricted outcome definition. In the high-completeness primary model population, 191 patients had the restricted outcome. The associated-factor pattern was generally consistent with that of the original composite CVD model. Age, male sex, and disease duration remained positively associated with the restricted outcome, whereas erythrocyte sedimentation rate remained non-significant. Detailed results are shown in Supplementary Table S6.

### 3.4 Machine learning analysis results

#### 3.4.1 Simplified conventional-risk baseline model

To provide a conventional-risk benchmark, a simplified logistic regression model using traditional cardiovascular risk factors was developed and evaluated on the same test set. The model included age, sex, hypertension, diabetes, dyslipidemia, smoking history, BMI, mean systolic blood pressure, and low-density lipoprotein cholesterol. Because formal cardiovascular risk scores could not be fully reconstructed due to unavailable or highly incomplete variables, this model was interpreted as a data-derived conventional-risk reference based on available variables rather than as a direct comparison with validated scores such as QRISK-3 or the Framingham Risk Score.

In the test set, the simplified conventional-risk baseline model without SMOTE achieved an AUC of 0.78 and a PR-AUC of 0.22. At the default threshold of 0.50, it showed high specificity (1.00) but very low sensitivity (0.04), indicating limited ability to identify patients with concurrent CVD under the default decision threshold. After applying a training-derived Youden threshold of 0.07, sensitivity increased to 0.68, whereas specificity decreased to 0.67. The SMOTE-based version of the conventional-risk model showed a similar AUC (0.78) and PR-AUC (0.22), with a sensitivity of 0.65 and an F1 score of 0.24 at the default threshold. The SMOTE-based core-variable logistic regression reference model showed a comparable AUC of 0.78, with higher sensitivity (0.75) and F1 score (0.27) at the default threshold. Detailed results are shown in Supplementary Table S7.

#### 3.4.2 Core-variable identification models

**Figure 1:**
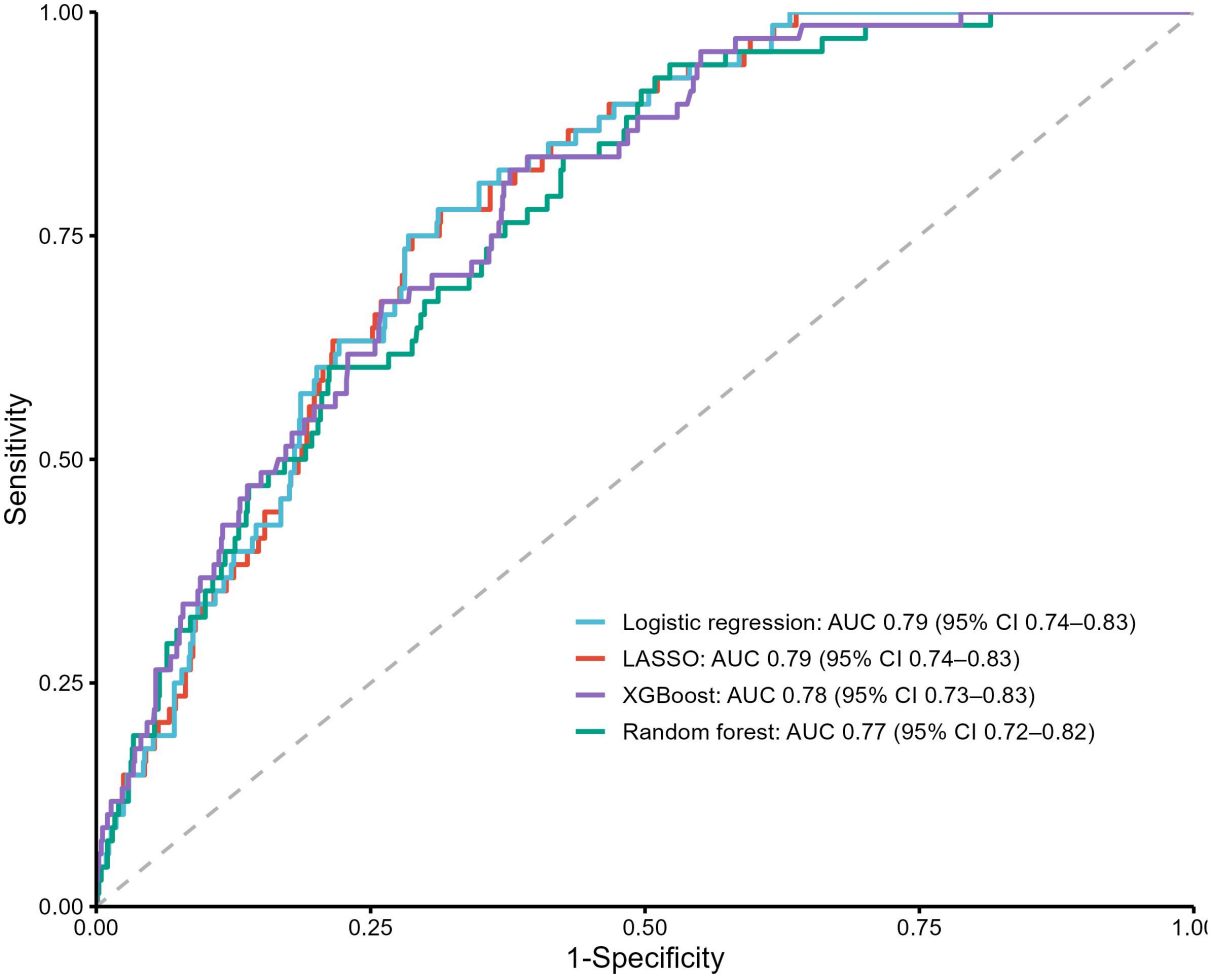
ROC curves of the core-variable identification models on the test set.

For comparison with the conventional-risk benchmark, core-variable identification models were further developed using nine clinically relevant variables: age, sex, disease duration, hypertension, diabetes, erythrocyte sedimentation rate, estimated glomerular filtration rate, white blood cell count, and hemoglobin. After the data were split into the training set and the test set at a ratio of 8:2, 3,813 patients were included in the training set and 954 patients were included in the test set. In the test set, 68 patients had RA-CVD and 886 had RA-non-CVD. Logistic regression, LASSO, random forest, and XGBoost were used for model development. The test-set performance is shown in Table 4, and the ROC curves are shown in Figure 1.

**Table 4:** Comparison of the performance of core-variable identification models on the test set.

Model	AUC	PR-AUC	Brier score	Accuracy	Sensitivity	Specificity	Positive predictive value	Negative predictive value	F1 score
Logistic	0.79	0.20	0.19	0.70	0.75	0.70	0.16	0.97	0.27
LASSO	0.79	0.20	0.19	0.70	0.75	0.70	0.16	0.97	0.26
Random Forest	0.77	0.19	0.18	0.78	0.53	0.80	0.17	0.96	0.26
XGBoost	0.78	0.24	0.20	0.65	0.74	0.64	0.14	0.97	0.23

The results showed that all four models had some discriminatory ability, with AUC values ranging from 0.77 to 0.79. Logistic regression and LASSO showed comparable discrimination, with both models achieving an AUC of 0.79. The AUCs of XGBoost and random forest were 0.78 and 0.77, respectively. For PR-AUC, the XGBoost model had the highest value (0.24), while the values for logistic regression, LASSO, and random forest were 0.20, 0.20, and 0.19, respectively. When classification performance was calculated using the default threshold of 0.50, the sensitivities of the logistic regression and LASSO models were both 0.75, which were higher than that of the random forest model (0.53). The sensitivity of the XGBoost model was 0.74, but its positive predictive value was relatively low.

In addition to discrimination and classification performance, model calibration was also evaluated using the Brier score and calibration curves. The Brier scores of the four core-variable models ranged from 0.18 to 0.20. The random forest model had the lowest Brier score (0.18), suggesting a relatively small overall probability estimation error in the test set.

#### 3.4.3 Wide-feature models and training-derived threshold adjustment

On the basis of the core-variable models, 86 automatically selected wide features were further included to build wide-feature models in order to evaluate the effect of richer structured variables on model identification performance. The test-set performance is shown in Table 5.

**Table 5:** Comparison of the performance of wide-feature identification models on the test set (default threshold = 0.50).

Model	AUC	PR-AUC	Brier score	Accuracy	Sensitivity	Specificity	Positive predictive value	Negative predictive value	F1 score
LASSO	0.84	0.31	0.06	0.93	0.07	1.00	1.00	0.93	0.14
Random Forest	0.81	0.27	0.06	0.93	0.00	1.00	^a^	0.93	^a^
XGBoost	0.84	0.32	0.06	0.93	0.06	1.00	1.00	0.93	0.11

The test set included 954 patients, including 68 with RA-CVD and 886 with RA-non-CVD. Classification performance metrics were calculated using the default threshold of 0.50.

a indicates that the model did not identify any positive cases at this threshold; therefore, the positive predictive value and F1 score could not be calculated.

Under the default threshold of 0.50, the AUCs of the wide-feature models ranged from 0.81 to 0.84, which were higher than those of the core-variable models. XGBoost and LASSO showed comparable discrimination, with both models achieving an AUC of 0.84, whereas random forest achieved an AUC of 0.81. XGBoost had the highest PR-AUC (0.32), followed by LASSO (0.31) and random forest (0.27). The three wide-feature models had similar Brier scores, all of which were 0.06 when presented to two decimal places. Although the wide-feature models showed high accuracy and specificity at the default threshold, their sensitivities were low. The sensitivities of LASSO and XGBoost were 0.07 and 0.06, respectively. Random forest had a sensitivity of 0.00 and did not identify any positive cases in the test set. These results indicate that, under marked class imbalance, the default threshold of 0.50 was unsuitable for identifying patients with concurrent CVD, despite acceptable discrimination by AUC.

Therefore, a training-derived Youden threshold was further determined based on the out-of-fold predicted probabilities from the training set, and the test-set performance after threshold adjustment was compared. The results are shown in Table 6. After exploratory threshold adjustment using training-derived out-of-fold probabilities, minority-class identification improved in all models. At the training-derived exploratory threshold, the LASSO model achieved an accuracy of 0.70, a sensitivity of 0.82, a specificity of 0.69, and an F1 score of 0.29. The random forest model achieved an accuracy of 0.73, a sensitivity of 0.74, a specificity of 0.73, and an F1 score of 0.28. The XGBoost model achieved an accuracy of 0.74, a sensitivity of 0.81, a specificity of 0.74, and an F1 score of 0.31. Considering AUC, PR-AUC, Brier score, and F1 score at the training-derived threshold, the XGBoost model showed the most favorable numerical profile in this internal test set. These internal results suggest that broader structured variables and training-derived threshold adjustment may improve minority-class identification, but the selected threshold requires external calibration before clinical use.

**Table 6:** Comparison of the performance of threshold-adjusted wide-feature models on the test set (training-derived Youden threshold).

Model	Training-derived Youden threshold	AUC	PR-AUC	Brier score	Accuracy	Sensitivity	Specificity	Positive predictive value	Negative predictive value	F1 score
LASSO	0.08	0.84	0.31	0.06	0.70	0.82	0.69	0.17	0.98	0.29
Random Forest	0.11	0.81	0.27	0.06	0.73	0.74	0.73	0.17	0.97	0.28
XGBoost	0.08	0.84	0.32	0.06	0.74	0.81	0.74	0.19	0.98	0.31

**Figure 2:**
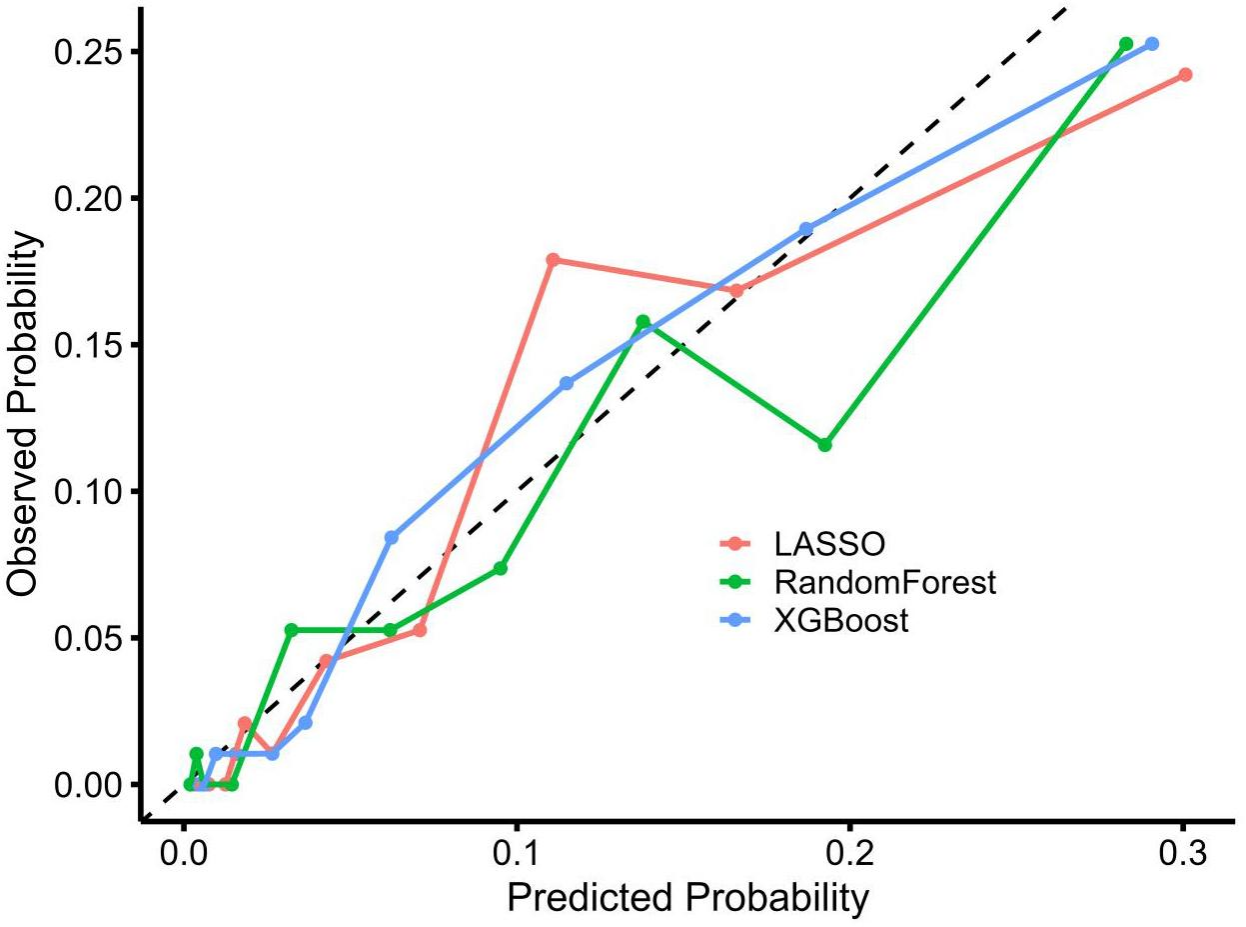
Calibration curves of the threshold-adjusted wide-feature models on the test set.

**Figure 3:**
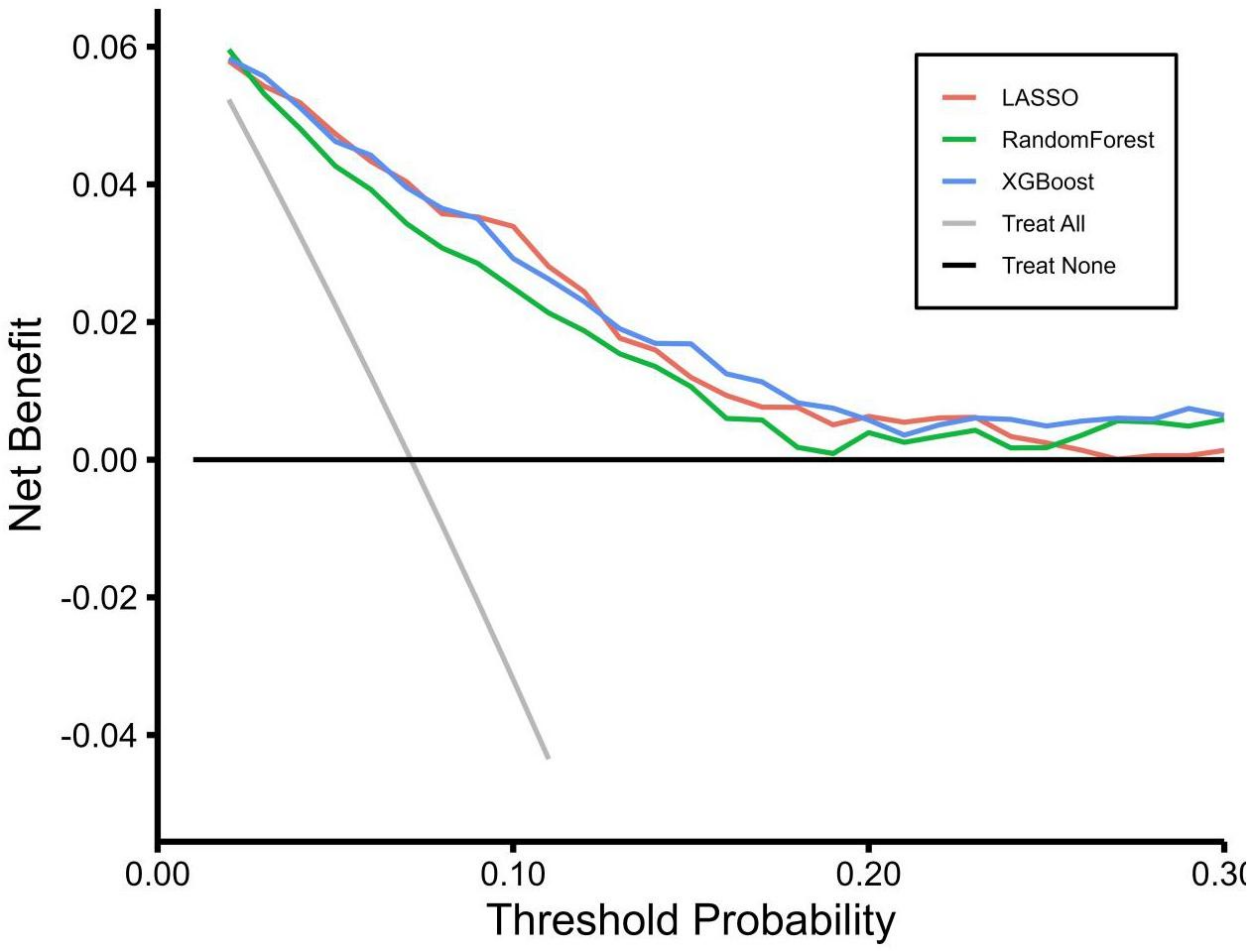
Decision curves of the threshold-adjusted wide-feature models on the test set.

In the threshold-adjusted wide-feature models, all three algorithms had Brier scores of 0.06 when presented to two decimal places. The calibration curves are shown in Figure 2. Overall, the calibration curves of the wide-feature models followed the ideal reference line to some extent, but visible deviations were present in several probability ranges. XGBoost showed relatively favorable visual calibration among the three models, although it slightly overestimated observed risk in the higher predicted-probability range. The LASSO model showed local fluctuation in the low-to-middle predicted-probability range, and the random forest model showed greater deviation in some middle-probability intervals. These findings suggest that the wide-feature models had acceptable but imperfect internal calibration, and that external calibration is still needed before clinical application. For the LASSO model, the Hosmer-Lemeshow test yielded a *P* value of 0.272, which suggested no obvious lack of fit. Decision curve analysis (Figure 3) suggested potential net benefit for the threshold-adjusted wide-feature models within the low-to-moderate threshold range, with the XGBoost model showing the most favorable numerical pattern among the compared models.

#### 3.4.4 Exploratory medication-information subgroup analysis

In patients with available medication information, a medication-enhanced analysis was further performed. This subgroup included 674 patients in total, with 538 in the training set and 136 in the test set. In the test set, 24 patients had RA-CVD and 112 had RA-non-CVD. The base feature set model included 9 variables, while the medication-enhanced model included 10 variables after adding one medication-related variable. The comparison of test-set performance is shown in Table 7.

**Table 7:** Comparison of test-set performance between the base models and the medication-enhanced models in the subgroup with available medication information.

Feature set	Model	AUC	PR-AUC	Brier score	Accuracy	Sensitivity	Specificity	Positive predictive value	Negative predictive value	F1 score
Base feature set	Logistic	0.77	0.39	0.18	0.71	0.54	0.75	0.32	0.88	0.40
	LASSO	0.77	0.40	0.18	0.71	0.63	0.73	0.33	0.90	0.43
	Random Forest	0.78	0.37	0.17	0.71	0.58	0.74	0.33	0.89	0.42
	XGBoost	0.74	0.29	0.18	0.72	0.58	0.75	0.33	0.89	0.42
Medication-enhanced feature set	Logistic	0.77	0.40	0.17	0.73	0.63	0.75	0.35	0.90	0.45
	LASSO	0.77	0.40	0.18	0.71	0.58	0.73	0.32	0.89	0.41
	Random Forest	0.78	0.34	0.17	0.73	0.54	0.77	0.33	0.89	0.41
	XGBoost	0.75	0.31	0.21	0.71	0.58	0.73	0.32	0.89	0.41

The test set of the subgroup with available medication information included 136 patients, including 24 with RA-CVD and 112 with RA-non-CVD. Classification performance metrics were calculated using the default threshold of 0.50.

In the base feature set models, the random forest model had the highest AUC (0.78). The AUCs of the LASSO, logistic regression, and XGBoost models were 0.77, 0.77, and 0.74, respectively. For PR-AUC, the LASSO and logistic regression models reached 0.40 and 0.39, respectively, which were higher than those of the random forest and XGBoost models. Under the default threshold of 0.50, the XGBoost model had the highest accuracy (0.72), while the LASSO, logistic regression, and random forest models all had an accuracy of 0.71.

In the medication-enhanced models, the AUC and accuracy of some algorithms changed slightly after the coarse-grained medication variable was added. Random forest achieved the highest AUC (0.78). Logistic regression and LASSO both achieved an AUC of 0.77, whereas XGBoost achieved an AUC of 0.75. Logistic regression and LASSO showed comparable PR-AUCs, both of which were 0.40 when presented to two decimal places. At the default threshold of 0.50, logistic regression and random forest both achieved an accuracy of 0.73, compared with 0.71 for LASSO and XGBoost.

## 4 Discussion

Based on single-center real-world clinical data, this study analyzed factors associated with concurrent CVD in patients with RA and further developed machine learning models for identification. The results showed clear differences between patients with RA-CVD and those with RA-non-CVD in age, sex distribution, disease duration, traditional cardiovascular risk factors, and several laboratory indicators. This finding is consistent with the general pattern reported in real-world studies. Patients with RA-CVD are usually older, have a heavier comorbidity burden, and show a less favorable overall clinical condition (17). A review on cardiovascular assessment in RA has pointed out that cardiovascular evaluation in RA should not simply follow the framework used for the general population, but should also consider traditional risk factors, inflammatory background, and disease burden (18). In this study, age, male sex, and disease duration showed good stability in the high-completeness primary model. Their effect directions and statistical significance were also generally consistent after multiple-imputation sensitivity analysis. These findings suggest that RA with CVD is better understood as a complex comorbidity state. It reflects the combined effects of baseline risk factors, long-term disease exposure, and chronic inflammatory burden (19). Although dyslipidemia is a clinically important cardiovascular comorbidity, it was not significantly associated with concurrent CVD in the univariate logistic regression analysis in this dataset. This may partly reflect incomplete recording of dyslipidemia history, treatment effects, and the lipid paradox described in RA populations.

From a mechanistic perspective, cardiovascular injury related to RA is not limited to increased atherosclerotic plaque burden. It also involves endothelial dysfunction, increased oxidative stress, immune cell recruitment, and microvascular remodeling. A review on endothelial dysfunction in rheumatic diseases showed that chronic inflammation can continuously damage vascular endothelial homeostasis by reducing nitric oxide bioavailability, increasing oxidative stress, and promoting the expression of adhesion molecules (20). On this basis, another review on coronary microvascular dysfunction in autoimmune rheumatic diseases further suggested that inflammation, endothelial injury, and autoimmunity can together lead to abnormal coronary microvascular perfusion. Therefore, cardiovascular involvement in RA may appear not only as large-vessel disease but also as earlier damage at the microvascular and myocardial levels (21). This mechanistic framework is consistent with the findings of the present study, in which age, disease duration, and inflammation-related indicators all pointed in the same direction for RA with CVD.

In this study, some inflammatory markers were associated with RA-CVD in the univariate analysis, but they did not remain independently significant in the multivariable models. This is in line with the multi-pathway nature of cardiovascular injury in RA. A review on the metabolic consequences of RA showed that patients with RA often have insulin resistance, metabolic syndrome, hypertension, and lipoprotein abnormalities. In this setting, the effect of inflammation usually does not appear as one isolated laboratory marker. Instead, it is reflected together with metabolic disturbance and organ dysfunction (22). In the present study, some renal, hematologic, and metabolic indicators showed changes in the same direction in the univariate analysis or in the extended-feature identification models. However, they did not show stable independent associations in the multivariable models. Together with previous studies, this suggests that these indicators may reflect a generally unfavorable physiological state or the cumulative effect of long-term inflammatory burden, rather than independent risk signals in this dataset. A large prospective registry study showed that higher RA disease activity was associated with faster decline in renal function. This suggests that renal dysfunction may be an important intermediate link between long-term inflammatory burden and cardiovascular risk (23). Lower hemoglobin showed a similar direction in this study. A study based on the NHANES database found that anemia in patients with RA was associated with higher risks of all-cause mortality and CVD mortality. Therefore, lower hemoglobin may also reflect a generally poor physiological state (24). The association between higher uric acid and RA-CVD also has a consistent pathological basis, because hyperuricemia may increase cardiovascular risk through amplification of inflammation, oxidative stress, and endothelial injury (25). Taken together, these findings suggest that RA with CVD is not simply marked by elevation of one inflammatory indicator. Instead, it reflects a composite phenotype with joint imbalance in inflammation, metabolism, renal function, and hematologic status.

In this study, CVD was treated as an overall outcome. This approach is consistent with the broad clinical spectrum of cardiovascular involvement related to RA. A recent cohort study showed that the overall risk of heart failure was increased in patients with RA, and this increase was mainly driven by heart failure with preserved ejection fraction (26). This finding suggests that cardiovascular injury in RA involves not only traditional ischemic events but also broader pathological processes, such as reduced myocardial compliance, abnormal microvascular perfusion, and impaired diastolic function. From the perspective of clinical management, a scoping review on routine cardiovascular risk assessment in RA pointed out that the current problem is not only insufficient awareness of risk. Another major problem is the lack of standardized, practical, and specialty-friendly assessment pathways (27). Therefore, the value of identifying associated factors in this study lies in clarifying the clinical profile of RA patients with concurrent CVD and in providing a variable framework for comorbidity-oriented cardiovascular assessment.

The restricted-outcome sensitivity analysis partly addressed the concern related to outcome heterogeneity by focusing on major atherosclerotic cardiovascular or cerebrovascular disease. The associated-factor pattern remained generally consistent with the primary composite-outcome analysis, suggesting that the main findings were not driven solely by the inclusion of more clinically heterogeneous CVD components such as heart failure/cardiac insufficiency. However, this analysis should be interpreted as a heterogeneity-reduction strategy rather than a complete subtype-specific analysis.

The simplified conventional-risk baseline model provided an interpretable internal reference based on available traditional cardiovascular risk factors, rather than a formal comparison with validated cardiovascular risk scores such as QRISK-3 or the Framingham Risk Score. Its discrimination was comparable to that of the core-variable logistic regression model, suggesting that conventional cardiovascular risk factors still captured a substantial part of the information related to concurrent CVD status in RA. However, at the default threshold of 0.50, the conventional-risk model had extremely low sensitivity, which again indicates that discrimination alone is insufficient for evaluating models under severe class imbalance. SMOTE improved minority-class identification by reducing the dominance of the majority class during training, but resampling also changed the class distribution seen by the model and may affect probability calibration. Therefore, the results should be interpreted using threshold-dependent metrics, calibration measures, and external validation rather than AUC alone.

The machine learning results showed that the core-variable models already had some discriminatory ability. After adding a broader set of structured variables, both AUC and PR-AUC improved further. This suggests that, beyond the few core factors retained in conventional statistical models, routine clinical data still contain additional information that can help identify the current CVD status. Among the different modeling strategies, the threshold-adjusted XGBoost model with wide features showed the most favorable numerical profile when AUC, PR-AUC, and F1 score were considered together. These findings suggest that broader structured variables and training-derived threshold adjustment may improve minority-class identification under imbalanced data conditions. A systematic review of artificial intelligence models for CVD pointed out that model evaluation should not rely only on AUC. It should also consider classification performance under class imbalance, classification thresholds, and the actual use scenario (28). The potential applicability of a model depends on classification threshold, calibration, and external generalizability, not only on ranking ability (29). In the present study, the low Youden threshold of 0.08 for the wide-feature XGBoost model should be interpreted within this framework. Because CVD accounted for only 7.68% of the cohort, the default threshold of 0.50 favored specificity but identified very few patients with concurrent CVD. After threshold adjustment, the XGBoost model achieved higher sensitivity (0.81), while specificity remained moderate (0.74) and the negative predictive value was high (0.98). Therefore, this threshold is better understood as a sensitivity-oriented triage threshold for identifying patients who may need further cardiovascular assessment, rather than as a stand-alone diagnostic cutoff. In such a use scenario, positive classifications should be followed by clinical review, medical history confirmation, and appropriate cardiovascular examination, which would help prevent inappropriate clinical decisions based solely on model output. Taken together, under the current single-center internal-validation setting, the threshold-adjusted wide-feature XGBoost model can be regarded as a numerically favorable candidate for concurrent CVD identification, whereas the core-variable models provide simpler reference models with greater interpretability. External validation and threshold calibration are required before any prospective clinical implementation.

The subgroup analysis with available medication information should be interpreted with particular caution. This subgroup included only 674 patients, and the test set contained only 24 patients with CVD. After adding a coarse-grained antirheumatic drug variable to the base feature set, the performance of some models changed slightly, but the overall gain was limited. Given the small number of positive test-set cases, these numerical differences may be unstable and should not be interpreted as evidence that medication information improved model performance. The current analysis was exploratory and was intended only to examine whether medication information might provide a preliminary signal of added value. Further studies using larger datasets with more complete medication exposure records, treatment duration, dosage, and drug-class information are needed to assess the contribution of medication variables more reliably.

This study still has several limitations. First, this was a cross-sectional study. The analysis identified factors associated with concurrent CVD and developed models for current comorbidity identification; it cannot establish temporality, support causal inference, or predict incident cardiovascular events. Therefore, all findings should be interpreted as relating to prevalent CVD comorbidity rather than future cardiovascular risk.

Second, external validation could not be performed in the present study. No independent RA-CVD cohort with comparable outcome definitions, variable structure, and data quality was available. In real-world clinical settings, external validation is challenging because diagnostic recording practices, laboratory panels, medication information, and missing-data patterns may differ across hospitals. Patient-level data sharing is also restricted by privacy, ethical, and institutional data-governance requirements. Therefore, the reported model performance and training-derived threshold settings should be regarded as internally validated findings. Their transportability to other clinical settings remains uncertain. Future research will continue to focus on external validation. Subject to research support and collaborative opportunities, we intend to collect independent RA cohorts through cooperation with other hospitals. Before validation, a common data dictionary will be used to harmonize eligibility criteria, CVD outcome definitions, variable coding, measurement units, and assessment time points across institutions. Because the availability of clinical variables may differ between hospitals, external validation will initially prioritize the core-variable models based on more routinely available predictors. The wide-feature models will be evaluated only in cohorts with sufficient coverage and compatible definitions of the required variables. The original fitted models, preprocessing procedures, and decision thresholds will then be applied without retraining, and discrimination, calibration, threshold-dependent performance, and clinical net benefit will be assessed. Any subsequent model updating or recalibration will be reported separately from the original external-validation results.

Third, several variables had substantial missingness. Missing rates exceeded 80% for smoking history, BMI, and blood pressure measurements. In routine clinical practice, these variables may be recorded more often in patients who receive more comprehensive evaluation. Patients with complete data may therefore differ systematically from those with incomplete data. Complete-case analysis may overrepresent such patients and reduce the representativeness of the analytic sample. The smaller sample size may also decrease the precision of effect estimates. Moreover, high missingness may distort the estimated associations of traditional cardiovascular factors and affect the performance of models containing these variables. The direction and magnitude of these biases could not be determined from the available data. To reduce their influence, data completeness was considered during model specification, and the primary model prioritized variables with greater availability. Multiple imputation was also performed as a sensitivity analysis. The results for age, male sex, and disease duration were generally consistent between the complete-case and imputed analyses, supporting the relative robustness of the main associated-factor pattern. Nevertheless, findings involving highly incomplete variables should be interpreted cautiously.

Fourth, the present study mainly focused on biomedical and routinely available clinical factors, including demographic characteristics, disease duration, comorbidities, physical measurements, and laboratory indicators. Non-biomedical factors, such as socioeconomic status, education, occupation, income, healthcare access, living environment, and other social determinants of health, were not available in the current dataset. These factors may further influence both RA and CVD and could add another level of complexity to comorbidity identification. Future studies should integrate biomedical variables with social determinants of health and other contextual information to develop more comprehensive machine learning models for identifying at-risk RA populations.

Finally, this study combined several cardiovascular and cerebrovascular diseases into one overall outcome. Although this approach helped characterize cardiovascular involvement in RA from a broad comorbidity perspective, it also introduced clinical heterogeneity. Different CVD subtypes may differ in pathophysiological mechanisms, associated-factor patterns, and clinical manifestations. Because of the limited event counts in several individual CVD subtypes and the non-mutually exclusive nature of outcome categories, subtype-specific models for each CVD category were not performed. Instead, a restricted-outcome sensitivity analysis focusing on major atherosclerotic cardiovascular or cerebrovascular disease was added. This analysis reduced, but did not eliminate, outcome heterogeneity, because coronary, cerebrovascular, and peripheral arterial diseases may still differ in pathophysiology and associated-factor patterns. Nevertheless, the main associated-factor pattern remained generally consistent. Further validation in larger datasets is still needed.

## 5 Conclusion

Based on real-world clinical data, this study analyzed factors associated with concurrent CVD in patients with RA and developed corresponding machine learning models for current comorbidity identification. The results showed that RA with CVD is a complex comorbidity state characterized by multiple associated demographic, disease-related, and laboratory factors. Among them, age, male sex, and longer disease duration showed relatively good stability in the multivariable analysis. Some traditional cardiovascular risk factors and laboratory indicators also provided additional information in the univariate analysis or the extended-feature identification models. The missing-data sensitivity analysis further supported the robustness of the high-completeness primary model. The restricted-outcome sensitivity analysis also showed a generally consistent associated-factor pattern when the outcome was limited to major atherosclerotic cardiovascular or cerebrovascular disease. The simplified conventional-risk baseline model showed that traditional cardiovascular risk factors retained considerable discriminatory information, but threshold-dependent sensitivity remained a major issue under class imbalance. In terms of model development, the core-variable models already showed some identification ability. After adding a broader range of structured variables and applying training-derived threshold adjustment, the overall model performance improved further. Among these models, the threshold-adjusted wide-feature XGBoost model showed the most favorable numerical profile in internal validation. This finding suggests that broader structured variables and training-derived threshold adjustment may improve minority-class identification in this imbalanced dataset, but this strategy requires external validation and calibration before clinical use.

These findings suggest that cardiovascular comorbidity in RA should be assessed from a comprehensive multi-factor perspective. Within the present single-center internally validated setting, clinical-data-based identification models may provide preliminary support for recognizing concurrent CVD comorbidity and prioritizing patients for further cardiovascular evaluation. However, these models should not be regarded as clinically transportable tools at this stage. A key limitation is that external validation could not be performed because an independent validation cohort was unavailable. Accordingly, the reported model performance and training-derived threshold settings should be interpreted as internally validated findings rather than externally generalizable estimates. Future multicenter prospective studies with more complete information on lifestyle, medication exposure, and follow-up outcomes are required to validate the associated factors, recalibrate decision thresholds, and assess model generalizability across clinical settings.

## Data Availability

The datasets analyzed in this study are not publicly available because patient-level data sharing is restricted by privacy, ethical, and institutional data-governance requirements. De-identified data may be available from the corresponding author on reasonable request, subject to approval by the relevant institution and applicable regulations.

## References

[1] Tian X Wang Q Jiang N Zhao Y Huang C Liu Y. Chinese Guidelines for the diagnosis and treatment of rheumatoid arthritis: 2024 update. Rheumatol Immunol Res. (2025) 5(4):189–208. 10.1515/rir-2024-0028 39802551 PMC11720473

[2] Zotova LA Enenkov NV. From joints to vessels: how rheumatoid arthritis therapy alters the fate of the heart. World J Cardiol. (2025) 17(9):109876. 10.4330/wjc.v17.i9.109876 41024972 PMC12476620

[3] Dijkshoorn B Raadsen R Nurmohamed MT. Cardiovascular disease risk in rheumatoid arthritis anno 2022. J Clin Med. (2022) 11(10):2704. 10.3390/jcm11102704 35628831 PMC9142998

[4] Drosos AA Venetsanopoulou AA Pelechas E Voulgari PV. Exploring cardiovascular risk factors and atherosclerosis in rheumatoid arthritis. Eur J Intern Med. (2024) 128:1–9. 10.1016/j.ejim.2024.07.016 39048336

[5] Weber BN Giles JT Liao KP. Shared inflammatory pathways of rheumatoid arthritis and atherosclerotic cardiovascular disease. Nat Rev Rheumatol. (2023) 19(7):417–28. 10.1038/s41584-023-00969-7 37231248 PMC10330911

[6] García-Gómez MC Padró T Muñoz-García N Bianchi M Álvarez L Badimon L. Dysfunctional antioxidant capacity of high-density lipoprotein in rheumatoid arthritis. Eur J Clin Invest. (2023) 53(8):e13999. 10.1111/eci.13999 36994808

[7] Karpouzas GA Papotti B Ormseth SR Palumbo M Hernandez E Adorni MP. Changes in serum cholesterol loading capacity are linked to coronary atherosclerosis progression in rheumatoid arthritis. RMD Open. (2024) 10(4):e004991. 10.1136/rmdopen-2024-004991 39719397 PMC11683967

[8] Weber B Weisenfeld D Massarotti E Seyok T Cremone G Lam E. Interplay between systemic inflammation, myocardial injury, and coronary microvascular dysfunction in rheumatoid arthritis: results from the LiiRA study. J Am Heart Assoc. (2024) 13(9):e030387. 10.1161/JAHA.123.030387 38686879 PMC11179857

[9] Hajiesmaeili Y Tamhankar P Stranges S Barra L. Factors associated with incident cardiovascular disease in patients with rheumatoid arthritis: a scoping review. Autoimmun Rev. (2024) 23(5):103539. 10.1016/j.autrev.2024.103539 38582291

[10] Hughes DM Coronado JIC Schofield P Yiu ZZN Zhao SS. The predictive accuracy of cardiovascular disease risk prediction tools in inflammatory arthritis and psoriasis: an observational validation study using the clinical practice research datalink. Rheumatology (Oxford). (2024) 63(12):3432–41. 10.1093/rheumatology/kead610 37966910 PMC11636560

[11] Asteris PG Douvika MG Karamani CA Skentou AD Chlichlia K Cavaleri L. A novel heuristic algorithm for the modeling and risk assessment of the COVID-19 pandemic phenomenon. Comput Model Eng Sci. (2020) 125(2):815–28. 10.32604/cmes.2020.013280

[12] Asteris PG Gandomi AH Armaghani DJ Kokoris S Papandreadi AT Roumelioti A. Prognosis of COVID-19 severity using DERGA, a novel machine learning algorithm. Eur J Intern Med. (2024) 125:67–73. 10.1016/j.ejim.2024.02.037 38458880

[13] Asteris PG Gandomi AH Armaghani DJ Tsoukalas MZ Gavriilaki E Gerber G. Genetic justification of COVID-19 patient outcomes using DERGA, a novel data ensemble refinement greedy algorithm. J Cell Mol Med. (2024) 28(4):e18105. 10.1111/jcmm.18105 38339761 PMC10863978

[14] Feng M Meng F Jia Y Wang Y Ji G Gao C. Exploration of risk factors for cardiovascular disease in patients with rheumatoid arthritis: a retrospective study. Inflammation. (2025) 48(4):1811–27. 10.1007/s10753-024-02157-5 39414673

[15] Zou YW Wu T Ouyang ZM. [Comparation of the performance of different cardiovascular risk models in predicting cardiovascular diseases risk among Chinese patients with rheumatoid arthritis]. Zhonghua Yi Xue Za Zhi. (2025) 105(41):3775–82. 10.3760/cma.j.cn112137-20250304-00514 41218894

[16] Shao Y Zhang H Shi Q Wang Y Liang Q. Clinical prediction models of rheumatoid arthritis and its complications: focus on cardiovascular disease and interstitial lung disease. Arthritis Res Ther. (2023) 25(1):159. 10.1186/s13075-023-03140-5 37658422 PMC10472585

[17] Tekeoglu S. Prevalence and risk factors of cardiovascular disease in rheumatoid arthritis patients: a comparative analysis of real-world data. Int J Gen Med. (2024) 17:5859–68. 10.2147/IJGM.S490916 39659512 PMC11630700

[18] Oiegar R Pop D. Cardiovascular risk assessment in patients with rheumatoid arthritis. J Clin Med. (2025) 14(18):6461. 10.3390/jcm14186461 41010664 PMC12470517

[19] Bedeković D Bošnjak I Bilić-Ćurčić I Kirner D Šarić S Novak S. Risk for cardiovascular disease development in rheumatoid arthritis. BMC Cardiovasc Disord. (2024) 24(1):291. 10.1186/s12872-024-03963-3 38834973 PMC11149346

[20] Husaini ASA Fathima A Halawa D Aakel N Erre GL Giordo R. Exploring endothelial dysfunction in major rheumatic diseases: current trends and future directions. J Mol Med (Berl). (2025) 103(6):635–49. 10.1007/s00109-025-02539-8 40229608 PMC12141126

[21] Cecere A Perazzolo Marra M Zanatta E Civieri G Iliceto S Tona F. Coronary microvascular dysfunction in autoimmune rheumatic diseases: beyond coronary flow velocity reserve. Front Cardiovasc Med. (2024) 11:1372703. 10.3389/fcvm.2024.1372703 39234606 PMC11371758

[22] Barry S Sheng E Baker JF. Metabolic consequences of rheumatoid arthritis. Arthritis Care Res (Hoboken). (2025) 77(10):1167–74. 10.1002/acr.25537 40176397 PMC12500507

[23] Fukui S Winkelmayer WC Tedeschi SK Marrugo J Guan H Harrold L. Disease activity of rheumatoid arthritis and kidney function decline: a large prospective registry study. Ann Rheum Dis. (2025) 84(2):201–9. 10.1136/ard-2024-226156 39919894 PMC11822225

[24] Dong J Yin X Zhang X Chen Z. Association between anemia and mortality in patients with rheumatoid arthritis: a retrospective cohort study of the national health and nutrition examination survey (NHANES) database. Prev Med Rep. (2025) 54:103068. 10.1016/j.pmedr.2025.103068 40336599 PMC12056958

[25] Kuwabara M Hisatome I Ae R Kosami K Aoki Y Andres-Hernando A. Hyperuricemia, a new cardiovascular risk. Nutr Metab Cardiovasc Dis. (2025) 35(3):103796. 10.1016/j.numecd.2024.103796 39939254

[26] Kawano Y Weber BN Weisenfeld D Jeffway MI Cai T McDermott GC. Risk of incident heart failure and heart failure subtypes in patients with rheumatoid arthritis. Arthritis Care Res (Hoboken). (2025) 77(5):631–9. 10.1002/acr.25481 39651569 PMC12040578

[27] Murphy L Saab MM Cornally N McHugh S Cotter P. Cardiovascular disease risk assessment in patients with rheumatoid arthritis: a scoping review. Clin Rheumatol. (2024) 43(7):2187–202. 10.1007/s10067-024-06996-3 38733423 PMC11189331

[28] Cai Y Cai Y-Q Tang L-Y Wang Y-H Gong M Jing T-C. Artificial intelligence in the risk prediction models of cardiovascular disease and development of an independent validation screening tool: a systematic review. BMC Med. (2024) 22(1):56. 10.1186/s12916-024-03273-7 38317226 PMC10845808

[29] Collins GS Dhiman P Ma J Schlussel MM Archer L Van Calster B. Evaluation of clinical prediction models (part 1): from development to external validation. BMJ. (2024) 384:e074819. 10.1136/bmj-2023-074819 38191193 PMC10772854

