# Supplementary material for: Factors associated with concurrent cardiovascular disease in patients with rheumatoid arthritis and development of machine learning models for comorbidity identification
Source: Front Cardiovasc Med. 2026 Aug 19;13:1860714. doi: 10.3389/fcvm.2026.1860714 (PMC13535289; doi:10.3389/fcvm.2026.1860714)
Supplement: Supplementary file 1 [file Datasheet1.docx]

Supplementary Material

# Supplementary Figures

Table S1. Composition of major CVD outcomes in the RA-CVD group

| CVD outcome type | Cases | Proportion (%) |
| --- | --- | --- |
| Coronary atherosclerotic disease-related | 214 | 58.47 |
| Stroke/cerebrovascular events | 105 | 28.69 |
| Heart failure/cardiac insufficiency | 13 | 3.55 |
| Peripheral arterial disease | 10 | 2.73 |
| Myocardial infarction | 5 | 1.37 |

Note: This table was summarized from diagnostic records and free-text previous medical history. Outcome categories are not mutually exclusive, and the same patient may have more than one CVD condition. Therefore, the sum of case counts may exceed the total number of 366 patients. All proportions were calculated using the total number of RA-CVD patients (n=366) as the denominator.

Table S2a. Missingness of major candidate clinical variables

| Variable | Non-missing n | Missing n | Missing rate (%) |
| --- | --- | --- | --- |
| Sex | 4,767 | 0 | 0.00 |
| Age | 4,767 | 0 | 0.00 |
| Disease duration | 4,164 | 603 | 12.65 |
| Westergren erythrocyte sedimentation rate, first hour | 2,886 | 1,881 | 39.46 |
| Blood glucose | 2,408 | 2,359 | 49.49 |
| Uric acid | 2,405 | 2,362 | 49.55 |
| Estimated glomerular filtration rate | 2,266 | 2,501 | 52.46 |
| Hypertension | 2,124 | 2,643 | 55.44 |
| Diabetes | 2,124 | 2,643 | 55.44 |
| Dyslipidemia | 2,124 | 2,643 | 55.44 |
| C-reactive protein | 1,495 | 3,272 | 68.64 |
| White blood cell count | 1,220 | 3,547 | 74.41 |
| Hemoglobin | 1,220 | 3,547 | 74.41 |
| Platelet count | 1,220 | 3,547 | 74.41 |
| BMI | 611 | 4,156 | 87.18 |
| Mean systolic blood pressure | 619 | 4,148 | 87.01 |
| Mean diastolic blood pressure | 619 | 4,148 | 87.01 |
| Smoking history | 603 | 4,164 | 87.35 |
| Rheumatoid factor | 398 | 4,369 | 91.65 |
| Neutrophil-to-lymphocyte ratio | 298 | 4,469 | 93.75 |
| Low-density lipoprotein cholesterol | 249 | 4,518 | 94.78 |
| Triglycerides | 213 | 4,554 | 95.53 |
| Total cholesterol | 211 | 4,556 | 95.57 |
| High-density lipoprotein cholesterol | 210 | 4,557 | 95.59 |
| Homocysteine | 103 | 4,664 | 97.84 |

Note: The full cohort included 4,767 patients. Missingness was calculated using the full cohort as the denominator. Variables are ordered approximately by increasing missingness. Variables with missing rates greater than 95% were excluded from the candidate set for the wide-feature machine learning models.

Table S2b. Inclusion of major candidate clinical variables in different analyses

| Variable | Univariate analysis | High-completeness primary model | Clinically enhanced model | Core-variable machine learning model | Candidate set for wide features |
| --- | --- | --- | --- | --- | --- |
| Sex | Yes | Yes | Yes | Yes | Yes |
| Age | Yes | Yes | Yes | Yes | Yes |
| Disease duration | Yes | Yes | Yes | Yes | Yes |
| Westergren erythrocyte sedimentation rate, first hour | Yes | Yes | Yes | Yes | Yes |
| Blood glucose | Yes | No | No | No | Yes |
| Uric acid | Yes | No | No | No | Yes |
| Estimated glomerular filtration rate | Yes | No | Yes | Yes | Yes |
| Hypertension | Yes | No | Yes | Yes | Yes |
| Diabetes | Yes | No | Yes | Yes | Yes |
| Dyslipidemia | Yes | No | No | No | Yes |
| C-reactive protein | Yes | No | No | No | Yes |
| White blood cell count | Yes | No | No | Yes | Yes |
| Hemoglobin | Yes | No | No | Yes | Yes |
| Platelet count | Yes | No | No | No | Yes |
| BMI | Yes | No | No | No | Yes |
| Mean systolic blood pressure | Yes | No | No | No | Yes |
| Mean diastolic blood pressure | Yes | No | No | No | Yes |
| Smoking history | Yes | No | No | No | Yes |
| Rheumatoid factor | Yes | No | No | No | Yes |
| Neutrophil-to-lymphocyte ratio | Yes | No | No | No | Yes |
| Low-density lipoprotein cholesterol | Yes | No | No | No | Yes |
| Triglycerides | Yes | No | No | No | No |
| Total cholesterol | Yes | No | No | No | No |
| High-density lipoprotein cholesterol | Yes | No | No | No | No |
| Homocysteine | Yes | No | No | No | No |

Note: “Yes” indicates inclusion in the corresponding analysis or candidate feature set. The high-completeness primary model prioritized variables with better availability and clinical relevance, whereas the clinically enhanced model additionally included clinically important variables with higher missingness. Variables with missing rates greater than 95% were excluded from the candidate set for the wide-feature machine learning models.

Table S3. List of 86 structured variables included in the wide-feature machine learning models

| No. | Variable name | Variable type | Missing rate (%) |
| --- | --- | --- | --- |
| 1 | Age | Numeric | 0.00 |
| 2 | Sex | Categorical | 0.00 |
| 3 | Visit type | Categorical | 0.00 |
| 4 | Disease duration | Numeric | 12.65 |
| 5 | Ethnicity | Categorical | 86.99 |
| 6 | Height | Numeric | 87.12 |
| 7 | Weight | Numeric | 87.18 |
| 8 | Smoking history | Numeric | 87.35 |
| 9 | Pain score | Numeric | 87.12 |
| 10 | Total activities of daily living score | Numeric | 91.29 |
| 11 | Grade | Categorical | 91.40 |
| 12 | First systolic blood pressure | Numeric | 87.01 |
| 13 | First diastolic blood pressure | Numeric | 87.01 |
| 14 | Second systolic blood pressure | Numeric | 88.02 |
| 15 | Second diastolic blood pressure | Numeric | 88.02 |
| 16 | Westergren erythrocyte sedimentation rate, first hour | Numeric | 39.46 |
| 17 | C-reactive protein | Numeric | 68.64 |
| 18 | White blood cell count | Numeric | 74.41 |
| 19 | Red blood cell count | Numeric | 74.41 |
| 20 | Hemoglobin | Numeric | 74.41 |
| 21 | Hematocrit | Numeric | 74.41 |
| 22 | Mean corpuscular volume | Numeric | 74.41 |
| 23 | Mean corpuscular hemoglobin | Numeric | 74.41 |
| 24 | Mean corpuscular hemoglobin concentration | Numeric | 74.41 |
| 25 | Red cell distribution width-SD | Numeric | 74.43 |
| 26 | Red cell distribution width-CV | Numeric | 74.43 |
| 27 | Platelet count | Numeric | 74.41 |
| 28 | Mean platelet volume | Numeric | 74.49 |
| 29 | Platelet distribution width | Numeric | 74.49 |
| 30 | Plateletcrit | Numeric | 74.49 |
| 31 | Large platelet ratio | Numeric | 74.49 |
| 32 | Neutrophil percentage | Numeric | 74.41 |
| 33 | Absolute neutrophil count | Numeric | 74.41 |
| 34 | Lymphocyte percentage | Numeric | 74.41 |
| 35 | Absolute lymphocyte count | Numeric | 74.41 |
| 36 | Monocyte percentage | Numeric | 74.41 |
| 37 | Absolute monocyte count | Numeric | 74.41 |
| 38 | Eosinophil percentage | Numeric | 74.41 |
| 39 | Absolute eosinophil count | Numeric | 74.41 |
| 40 | Basophil percentage | Numeric | 74.41 |
| 41 | Absolute basophil count | Numeric | 74.41 |
| 42 | Rheumatoid factor | Numeric | 91.65 |
| 43 | Immature granulocyte percentage | Numeric | 93.75 |
| 44 | Nucleated red blood cell percentage | Numeric | 93.75 |
| 45 | Neutrophil-to-lymphocyte ratio | Numeric | 93.75 |
| 46 | High-fluorescence lymphocytes | Numeric | 93.75 |
| 47 | Alanine aminotransferase | Numeric | 53.39 |
| 48 | Lactate dehydrogenase | Numeric | 68.28 |
| 49 | Carbon dioxide combining power | Numeric | 49.55 |
| 50 | Estimated glomerular filtration rate | Numeric | 52.46 |
| 51 | Low-density lipoprotein cholesterol | Numeric | 94.78 |
| 52 | Prealbumin | Numeric | 58.38 |
| 53 | Aspartate aminotransferase | Numeric | 53.39 |
| 54 | Urea | Numeric | 49.53 |
| 55 | Uric acid | Numeric | 49.55 |
| 56 | Total bile acids | Numeric | 73.53 |
| 57 | Total bilirubin | Numeric | 53.49 |
| 58 | Total protein | Numeric | 53.49 |
| 59 | Chloride | Numeric | 51.44 |
| 60 | Hemolysis | Numeric | 94.40 |
| 61 | Globulin | Numeric | 53.49 |
| 62 | Albumin | Numeric | 53.49 |
| 63 | Albumin/globulin ratio | Numeric | 53.49 |
| 64 | Direct bilirubin | Numeric | 68.32 |
| 65 | Alkaline phosphatase | Numeric | 68.26 |
| 66 | Phosphorus | Numeric | 68.43 |
| 67 | Creatinine | Numeric | 49.53 |
| 68 | Creatine kinase | Numeric | 93.43 |
| 69 | Cholinesterase | Numeric | 53.49 |
| 70 | Cystatin C | Numeric | 74.62 |
| 71 | Lipemia | Numeric | 94.40 |
| 72 | Blood glucose | Numeric | 49.49 |
| 73 | Complement C1q | Numeric | 74.60 |
| 74 | Gamma-glutamyl transferase | Numeric | 67.95 |
| 75 | Calcium | Numeric | 68.39 |
| 76 | Sodium | Numeric | 51.44 |
| 77 | Potassium | Numeric | 51.44 |
| 78 | Icterus | Numeric | 94.40 |
| 79 | Ordered grade | Numeric | 91.40 |
| 80 | BMI | Numeric | 87.18 |
| 81 | Mean systolic blood pressure | Numeric | 87.01 |
| 82 | Mean diastolic blood pressure | Numeric | 87.01 |
| 83 | History documented | Numeric | 0.00 |
| 84 | Hypertension | Numeric | 55.44 |
| 85 | Diabetes | Numeric | 55.44 |
| 86 | Dyslipidemia | Numeric | 55.44 |

Table S4. Comparison between complete-case and multiple-imputation results for the high-completeness primary model

| Variable | Complete-case OR (95% CI) | P value | MI OR (95% CI) | P value | Direction consistency |
| --- | --- | --- | --- | --- | --- |
| Age (per 1-year increase) | 1.07 (1.06–1.09) | <0.001 | 1.08 (1.07–1.09) | <0.001 | Yes |
| Sex: Male vs Female | 2.20 (1.63–2.98) | <0.001 | 1.72 (1.35–2.18) | <0.001 | Yes |
| Disease duration (per 1-year increase) | 1.02 (1.01–1.04) | 0.006 | 1.01 (1.00–1.03) | 0.026 | Yes |
| Erythrocyte sedimentation rate (per 10-mm/h increase) | 1.01 (0.97–1.06) | 0.570 | 1.01 (0.96–1.06) | 0.642 | Yes |

Note: MI, multiple imputation; OR, odds ratio; CI, confidence interval. The OR for erythrocyte sedimentation rate is reported per 10-mm/h increase; age and disease duration are reported per 1-year increase. ORs and 95% CIs are presented to two decimal places, whereas P values are presented to three decimal places. Direction consistency was assessed using the unrounded regression coefficients. Statistical inference was based on unrounded estimates; therefore, a confidence-interval boundary displayed as 1.00 may correspond to an unrounded value slightly above or below 1.00. In the high-completeness primary model, the effect directions of all variables were consistent between the complete-case and multiple-imputation analyses.

Table S5. Comparison between complete-case and multiple-imputation results for the clinically enhanced model

| Variable | Complete-case OR (95% CI) | P value | MI OR (95% CI) | P value | Direction consistency |
| --- | --- | --- | --- | --- | --- |
| Age (per 1-year increase) | 1.06 (1.03–1.08) | <0.001 | 1.08 (1.06–1.09) | <0.001 | Yes |
| Sex: Male vs Female | 2.26 (1.52–3.35) | <0.001 | 1.72 (1.35–2.19) | <0.001 | Yes |
| Disease duration (per 1-year increase) | 1.01 (0.99–1.03) | 0.459 | 1.02 (1.00–1.03) | 0.022 | Yes |
| Hypertension: Yes vs No | 0.90 (0.60–1.35) | 0.612 | 1.02 (0.78–1.34) | 0.873 | No |
| Diabetes: Yes vs No | 0.79 (0.43–1.44) | 0.447 | 1.11 (0.76–1.61) | 0.578 | No |
| Erythrocyte sedimentation rate (per 10-mm/h increase) | 1.04 (0.98–1.11) | 0.231 | 1.01 (0.96–1.06) | 0.704 | Yes |
| Estimated glomerular filtration rate (per 10-mL/min/1.73 m² increase) | 0.99 (0.87–1.13) | 0.883 | 0.96 (0.85–1.08) | 0.485 | Yes |

Note: MI, multiple imputation; OR, odds ratio; CI, confidence interval. The OR for erythrocyte sedimentation rate is reported per 10-mm/h increase, and the OR for estimated glomerular filtration rate is reported per 10-mL/min/1.73 m² increase. Age and disease duration are reported per 1-year increase. ORs and 95% CIs are presented to two decimal places, whereas P values are presented to three decimal places. Direction consistency was assessed using the unrounded regression coefficients. Statistical inference was based on unrounded estimates; therefore, a confidence-interval boundary displayed as 1.00 may correspond to an unrounded value slightly above or below 1.00. Age and sex remained consistently associated with concurrent CVD in the complete-case and multiple-imputation analyses. The statistical significance of disease duration differed between the two analyses, whereas the effect directions of hypertension and diabetes were inconsistent.

Table S6. Restricted-outcome sensitivity analysis using major atherosclerotic cardiovascular or cerebrovascular disease as the outcome

| Outcome definition | N | Events | Variable | OR (95% CI) | P value |
| --- | --- | --- | --- | --- | --- |
| Composite CVD | 2629 | 219 | Age (per 1-year increase) | 1.07 (1.06–1.09) | <0.001 |
| Composite CVD | 2629 | 219 | Male sex | 2.20 (1.63–2.98) | <0.001 |
| Composite CVD | 2629 | 219 | Disease duration (per 1-year increase) | 1.02 (1.01–1.04) | 0.006 |
| Composite CVD | 2629 | 219 | Erythrocyte sedimentation rate (per 10-mm/h increase) | 1.01 (0.97–1.06) | 0.570 |
| Restricted vascular outcome | 2629 | 191 | Age (per 1-year increase) | 1.08 (1.06–1.09) | <0.001 |
| Restricted vascular outcome | 2629 | 191 | Male sex | 2.34 (1.70–3.22) | <0.001 |
| Restricted vascular outcome | 2629 | 191 | Disease duration (per 1-year increase) | 1.02 (1.00–1.03) | 0.018 |
| Restricted vascular outcome | 2629 | 191 | Erythrocyte sedimentation rate (per 10-mm/h increase) | 1.02 (0.97–1.08) | 0.353 |

Note: Composite CVD refers to the original composite cardiovascular disease outcome used in the main analysis. The restricted vascular outcome included coronary atherosclerotic disease-related diagnoses, myocardial infarction, stroke/cerebrovascular events, and peripheral arterial disease. Heart failure/cardiac insufficiency was excluded because of its greater clinical and pathophysiological heterogeneity. This analysis was intended to reduce, but not eliminate, heterogeneity in the composite CVD outcome. The OR for erythrocyte sedimentation rate is reported per 10-mm/h increase; age and disease duration are reported per 1-year increase. ORs and 95% CIs are presented to two decimal places, whereas P values are presented to three decimal places. Statistical inference was based on unrounded estimates. OR, odds ratio; CI, confidence interval.

Table S7. Test-set comparison of the simplified conventional-risk baseline models and the core-variable logistic regression reference model

| Model | Threshold | AUC | PR-AUC | Sens | Spec | F1 | Brier |
| --- | --- | --- | --- | --- | --- | --- | --- |
| Conventional-risk LR, no SMOTE | 0.50 | 0.78 | 0.22 | 0.04 | 1.00 | 0.08 | 0.06 |
| Conventional-risk LR, no SMOTE | Youden 0.07 | 0.78 | 0.22 | 0.68 | 0.67 | 0.23 | 0.06 |
| Conventional-risk LR, SMOTE | 0.50 | 0.78 | 0.22 | 0.65 | 0.71 | 0.24 | 0.19 |
| Conventional-risk LR, SMOTE | Youden 0.52 | 0.78 | 0.22 | 0.63 | 0.73 | 0.25 | 0.19 |
| Core-variable LR, SMOTE | 0.50 | 0.78 | 0.20 | 0.75 | 0.70 | 0.27 | 0.19 |
| Core-variable LR, SMOTE | Youden 0.42 | 0.78 | 0.20 | 0.82 | 0.62 | 0.24 | 0.19 |

Note: LR, logistic regression; SMOTE, synthetic minority over-sampling technique; AUC, area under the receiver operating characteristic curve; PR-AUC, area under the precision-recall curve; Sens, sensitivity; Spec, specificity; F1, F1 score. The simplified conventional-risk baseline model included age, sex, hypertension, diabetes, dyslipidemia, smoking history, BMI, mean systolic blood pressure, and low-density lipoprotein cholesterol. Formal QRISK-3 and Framingham Risk Score models could not be directly reconstructed because several required variables were unavailable or highly incomplete. Youden thresholds were derived from out-of-fold predictions in the training set and then applied to the test set. Model-performance estimates and decision thresholds are presented to two decimal places.
